# Renal cell carcinoma in a child presenting with chronic gross hematuria: A rare case report with diagnostic and surgical insights

**DOI:** 10.1016/j.eucr.2026.103586

**Published:** 2026-08-21

**Authors:** Agung Damai Harikatang, Safendra Siregar, Ghani Ikhsan Majid

**Affiliations:** Department of Urology Hasan Sadikin General Hospital, Bandung, Indonesia

**Keywords:** Pediatric, Renal cell carcinoma, Gross hematuria, Renal tumor, Radical nephrectomy

## Abstract

Renal cell carcinoma is the most common adult renal malignancy but is rare in children, where Wilms tumor predominates. A 10-year-old boy presented with intermittent gross hematuria for two years, worsening with clot retention. Imaging revealed a right renal solid mass without metastasis (cT1bN0M0). After cystoscopic clot evacuation, open radical nephrectomy was performed. Histopathology confirmed RCC with lymphovascular invasion and negative margins (pT2aN0Mx). Because clinical and radiologic findings overlap with other pediatric renal tumors, definitive diagnosis relies on histopathology. Surgical resection is the primary treatment, as pediatric RCC responds poorly to chemo-and radiotherapy. Early-stage disease generally has favorable outcomes.

## 1 Introduction

Renal cell carcinoma (RCC) is a malignant neoplasm originating from the renal tubular epithelium and represents the most common form of kidney cancer in adults, accounting for approximately 85% of primary renal malignancies worldwide. However, RCC in the pediatric population is rare, constituting only a small fraction of childhood renal tumors and posing significant diagnostic and therapeutic challenges. Unlike adults, in whom RCC comprises the majority of renal cancers and is increasingly detected incidentally, children more commonly develop Wilms tumor, a distinct embryonal malignancy that predominates in early childhood. Consequently, RCC is often not the initial clinical suspicion in pediatric patients presenting with renal masses. Accurate diagnosis of RCC in pediatric patients is critical due to its distinct biological behavior and differing management strategies compared to other pediatric renal tumors. Radiological evaluation with ultrasonography and contrast-enhanced computed tomography facilitates lesion characterization and staging, but definitive diagnosis ultimately requires histopathological confirmation.^1^^,^^2^

The standard treatment for localized RCC, including in pediatric patients, remains surgical resection, as pediatric RCC has historically demonstrated limited responsiveness to conventional chemotherapy and radiotherapy. Radical nephrectomy offers the best chance for cure in cases of localized disease, and nephron-sparing approaches may be considered in select patients with small, peripherally located tumors to preserve renal function.^1^^,^^2^ Given the rarity of pediatric RCC and the consequent scarcity of high-level evidence, individual case reports continue to play an important role in expanding clinical understanding, refining diagnostic strategies, and informing surgical decision-making. This case report describes a 10-year-old boy with chronic gross hematuria found to have a right renal mass, ultimately diagnosed as RCC, and highlights pertinent considerations in differential diagnosis, imaging interpretation, and definitive management in the pediatric population.

## 2 Case presentation

A 10-year-old boy was referred to the urology department with a chief complaint of gross hematuria. The patient had experienced intermittent red-colored urine for approximately two years prior to presentation, with progressive worsening over the preceding two days characterized by the passage of blood clots during micturition. There was no associated flank pain, abdominal distension, dysuria, fever, dyspnea, lower limb edema, or altered level of consciousness. The patient denied a history of urinary tract stones, cloudy urine, or sandy urine. There was no prior history of hypertension, diabetes mellitus, chronic kidney disease, hemodialysis, or previous surgical procedures. The daily urine output was approximately 1000 mL, with persistent hematuria. Physical examination demonstrated a flat and soft abdomen with normal bowel sounds and no tenderness. No palpable renal masses were detected bilaterally, and there was no costovertebral angle tenderness. Suprapubic examination revealed an empty bladder without tenderness. External genital examination was unremarkable, with no evidence of meatal stenosis.

**Fig. 1:**
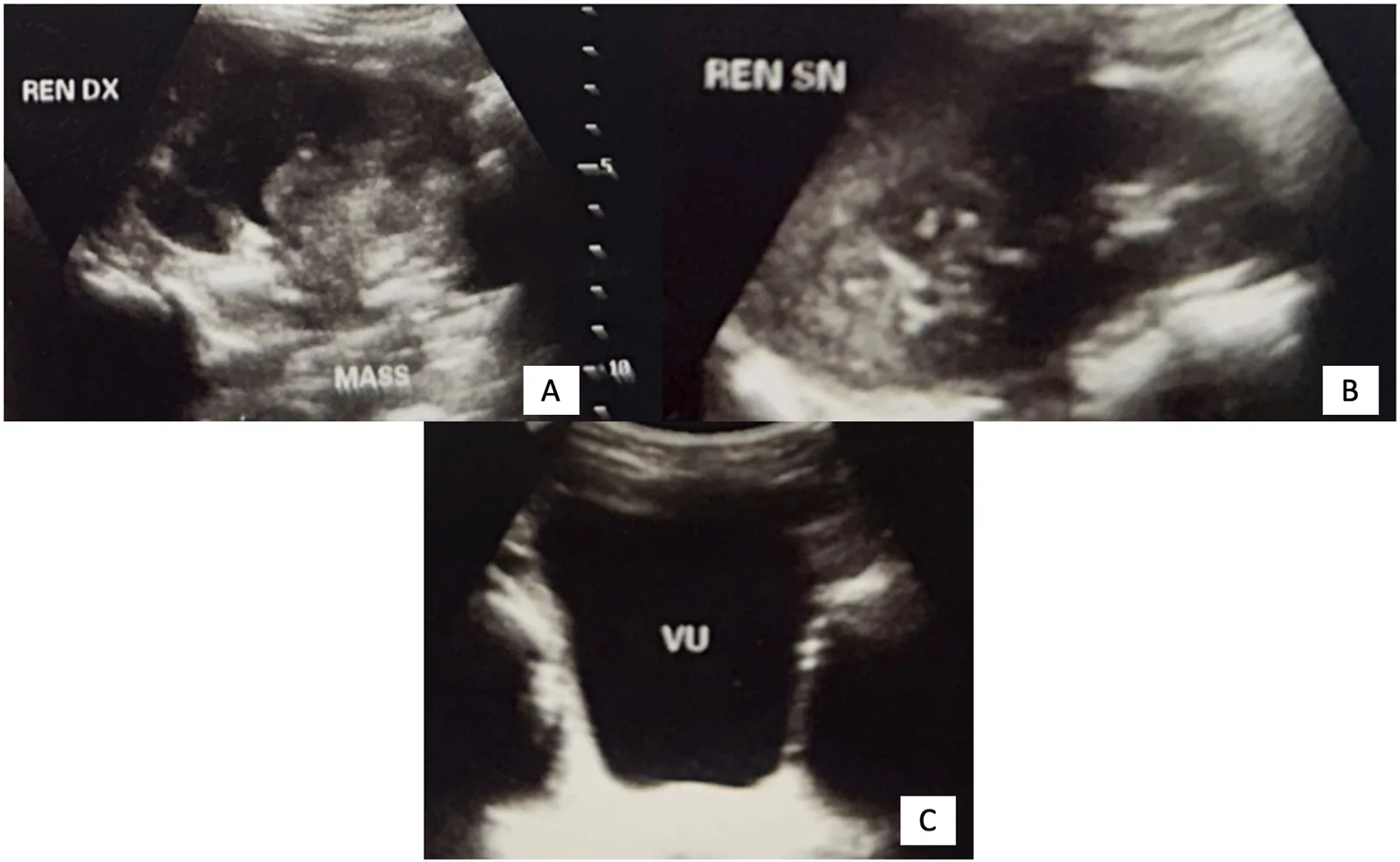
Ultrasonographic examination of right kidney (A), left kidney (B), and bladder (C). This findings showed mass on the right kidney.

**Fig. 2:**
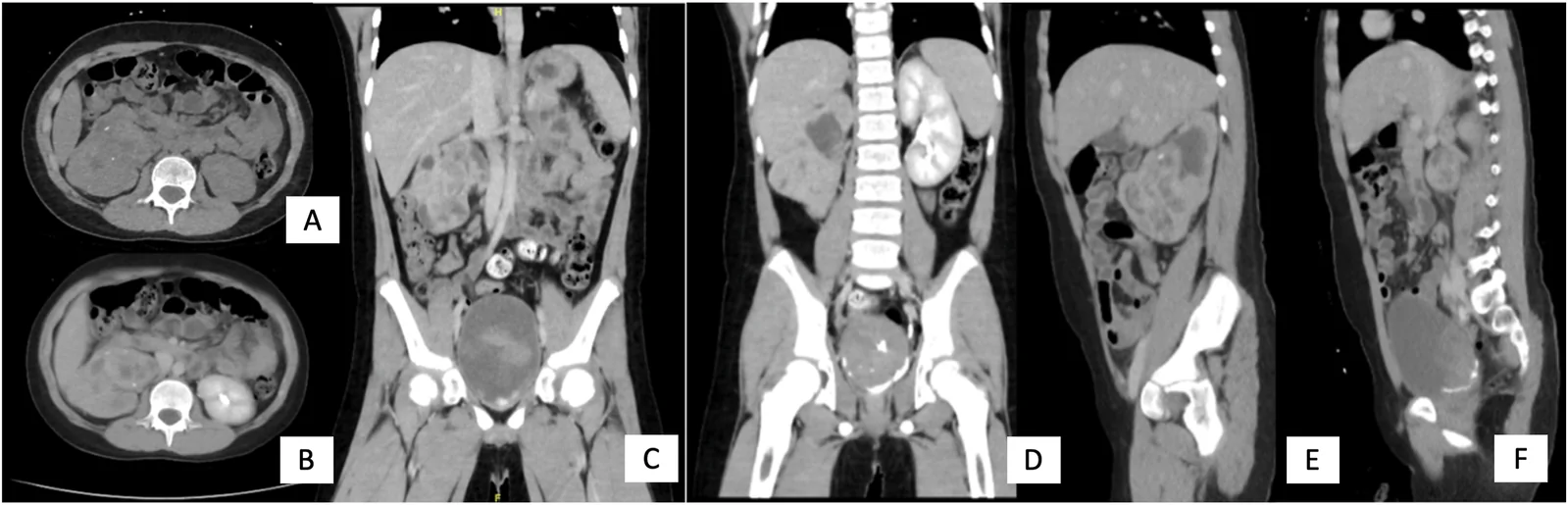
Non-contrast abdominal CT scan. Pre-contrast transverse section (A), post-contrast transverse section, coronal section (C-D), sagittal section (E-F). This finding showed mass on the right kidney with suspected of Wilms tumor.

Prior to referral, an ultrasonographic examination performed at a secondary healthcare facility revealed a mass in the right kidney (Fig. 1). The patient was subsequently referred for further evaluation and definitive management. On admission, the patient was conscious and alert, with a Karnofsky performance status score of 90. Vital signs were within normal limits, with a blood pressure of 98/69 mmHg, heart rate of 100 beats per minute, respiratory rate of 20 breaths per minute, body temperature of 37.3°C, and oxygen saturation of 98% on room air. Contrast-enhanced computed tomography (CT) of the abdomen and pelvis revealed a solid mass arising from the right kidney, consistent with a renal neoplasm. There was no evidence of regional lymph node enlargement or distant metastasis (Fig. 2). Chest radiography showed no pulmonary lesions. Based on radiological findings, the tumor was clinically staged as cT1bN0M0. During hospitalization, the patient developed urinary retention secondary to intravesical blood clots.

Surgical intervention was performed under general anesthesia with therapeutic intent. Cystoscopy was initially conducted with the patient in the lithotomy position. The external urethral meatus and urethra were normal. The bladder neck was not elevated, and no obstructive features were identified. Approximately 100 mL of intravesical blood clots were found and evacuated. The bladder mucosa appeared hyperemic, without evidence of trabeculation, sacculation, diverticula, calculi, or intravesical masses. A 14 Fr three-way Foley catheter was inserted, and continuous bladder irrigation was initiated.

The patient was subsequently repositioned supine, and an open right radical nephrectomy was performed via a chevron incision using a transperitoneal approach. Intraoperatively, the right kidney was enlarged to approximately 1.5 times the normal size and exhibited adhesions to surrounding tissues; however, it was successfully mobilized. The right ureter was similarly enlarged and adherent but could be dissected free. Radical nephrectomy was completed without complication, followed by meticulous hemostasis. Of note, a regional lymph node dissection or sampling was not performed during the procedure, as there was no intraoperative or radiologic evidence of lymphadenopathy. A silicone drain was placed, and the surgical wound was closed in layers. The total operative time was approximately 2 h, with an estimated blood loss of 250 mL. No intraoperative complications occurred.

**Fig. 3:**
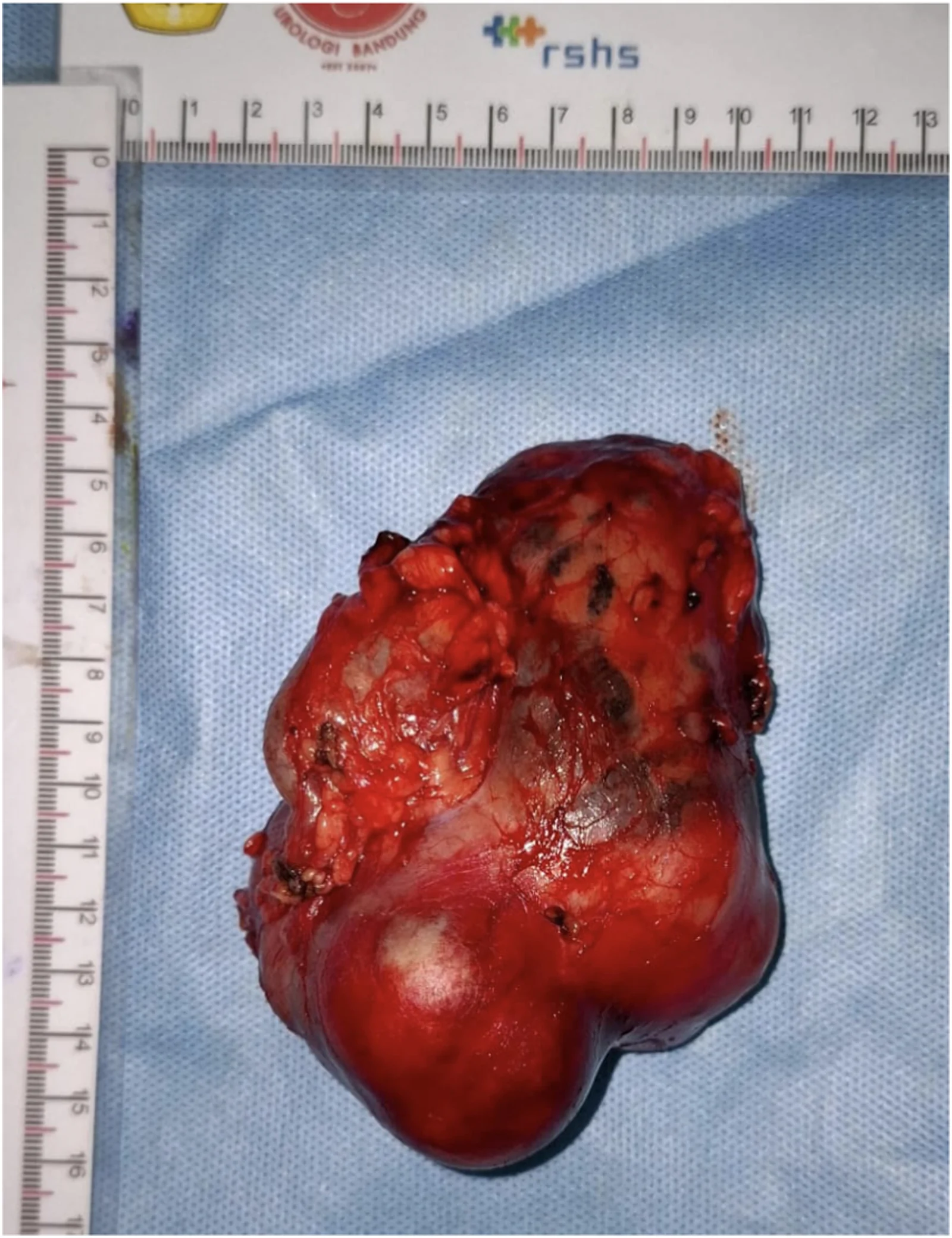
Gross appearance of the resected tumor and right kidney.

Gross pathological examination of the nephrectomy specimen revealed a kidney weighing 174 g and measuring 11 × 7.5 × 5 cm. A well-defined, soft, whitish-brown mass measuring approximately 9 × 6 × 2.5 cm was identified, with areas of hemorrhagic cystic degeneration. The tumor was confined to the renal parenchyma and did not extend beyond the renal capsule. The renal pelvis was compressed by the mass but showed no evidence of tumor invasion. The ureteral margin appeared grossly unremarkable (Fig. 3).

**Fig. 4:**
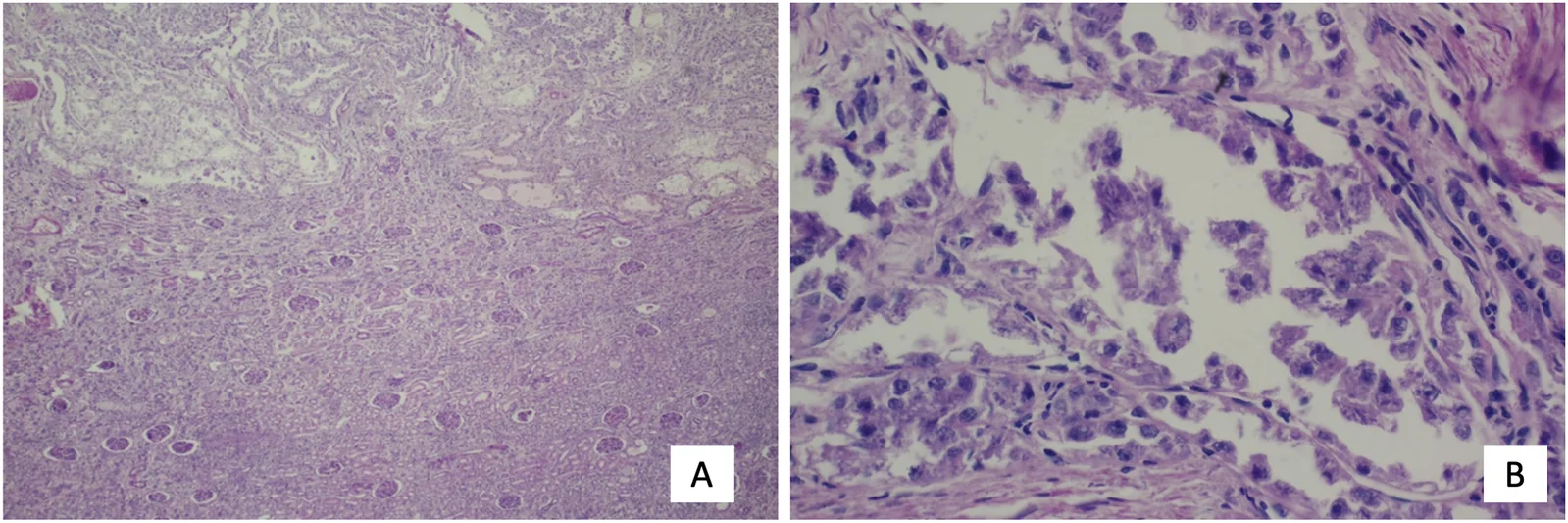
Histopathological examination of tumor. (A) showing the malignant and benign cell layer with transitional cell, (B) showing malignant cell.

Microscopically, the tumor was composed of cells arranged in alveolar, papillary, cystic, and focal solid patterns, separated by fibrous stroma infiltrated by lymphocytes. The tumor cells exhibited marked pleomorphism, hyperchromatic nuclei with occasional prominent nucleoli, abundant eosinophilic cytoplasm, and frequent mitotic figures. Areas of stromal hemorrhage were observed (Fig. 4). Based on these findings, the final histopathological diagnosis was renal cell carcinoma of the right kidney, staged as pT2aN0Mx, with evidence of lymphovascular invasion and no involvement of adjacent structures. The patient was discharged on the 3rd day after surgery, as expected, while the catheter was removed 7 days later. The patient is behaving well after 6 months of follow up visits with no adverse outcomes.

## 3 Discussion

Renal cell carcinoma (RCC) is an uncommon malignancy in the pediatric population, representing only a small proportion of childhood renal tumors. RCC accounts for approximately 1.4–6.3% of renal tumors in children, with incidence increasing considerably in older age groups, particularly after 10 years of age. RCC represented more than half of renal malignancies in adolescents aged 10–15 years, highlighting the age-dependent epidemiology that distinguishes pediatric RCC from both early-onset Wilms tumor and adult RCC.^3^^,^^4^

RCC in children frequently presents with clinical symptoms such as gross hematuria, flank or abdominal pain, and occasionally a palpable mass, similar to the presentation in our patient who experienced chronic hematuria with blood clot retention. While hematuria may be intermittent and nonspecific, it often prompts imaging which leads to further evaluation.^5^^,^^6^ Craig et al. has reported hematuria as one of the most reproducible presenting symptoms, even in the absence of other signs, underscoring the importance of considering RCC in differential diagnosis when persistent hematuria occurs without other clear causes. Importantly, children with RCC tend to present symptomatically and with larger tumors at diagnosis compared to adult counterparts who may have incidental lesions discovered on imaging.^7^

Differential diagnosis is pivotal in pediatric renal masses due to the high prevalence of Wilms tumor, which intuitively comprises the majority of renal tumors in children under 5 years old and follows different therapeutic algorithms involving neoadjuvant chemotherapy and specific radiotherapy protocols.^8^ Unlike Wilms tumor, RCC is typically chemotherapy and radiotherapy resistant, necessitating early surgical resection rather than preoperative chemotherapy, as highlighted in rare reports of concomitant RCC and Wilms tumor where chemotherapy failed to reduce tumor burden.^9^

In imaging studies, characteristics such as calcifications, cystic components, and heterogeneous enhancement may suggest RCC rather than Wilms tumor and influence the decision against preoperative biopsy due to the risk of needle tract seeding.^3^ However, imaging overlap exists among pediatric renal malignancies; clear cell sarcoma of the kidney and malignant rhabdoid tumors share nonspecific radiologic features. Hence, definitive diagnosis continues to rely on histopathology and immunohistochemistry, a practice echoed in multiple case series.^10^ Pediatric RCC also demonstrates a distinct genetic and morphologic profile compared to adult RCCs, with translocation-associated subtypes (Xp11.2/TFE3) reported in 40–50% of cases. These genetic alterations may not only influence histologic patterns but also have emerging implications for targeted therapies and prognosis.^5^

Initial diagnostic evaluation typically includes ultrasonography as a screening tool, followed by contrast-enhanced computed tomography (CECT) to better define tumor extent, enhancement patterns, and local staging, as performed in our case. While MRI can provide additional soft-tissue characterization, CT remains the mainstay in acute evaluation due to availability and speed. Functional imaging (PET) may have additional roles in select cases assessing metabolic activity and metastasis.^11^ Shaeh et al. has also explored CT radiomics and machine learning approaches to differentiate among pediatric renal tumors, though these methodologies are not yet standard practice and require further validation.^3^

Complete surgical resection remains the cornerstone of management for localized pediatric RCC due to the inherent resistance of RCC to conventional chemotherapy and radiotherapy. In a Khondker et al. systematic review, nephron-sparing surgery (NSS) was feasible in select patients with smaller tumor size and offers a potential benefit in preserving renal function; however, the evidence remains limited due to small sample sizes and selection bias. Our decision to proceed with open right radical nephrectomy reflects current consensus for localized tumors with significant size or suspected invasion, and is supported by international experience indicating surgical resection as the principal curative intervention. However, an important consideration in the surgical management of pediatric renal masses, including RCC is the role of regional lymph node sampling (LNS) or lymph node dissection (LND). Current surgical protocols from both the Children's Oncology Group (COG) and the International Society of Paediatric Oncology (SIOP) mandate that lymph node sampling be routinely performed at the time of radical nephrectomy, regardless of whether the nodes appear radiologically or intraoperatively normal. The COG protocol, for instance, emphasizes that LNS is essential to confirm a true node-negative status, establishing a target template that includes the renal hilar, paraaortic, and/or paracaval nodes. Despite these guidelines, the omission or low yield of lymph nodes remains the most frequent surgical protocol violation in pediatric renal surgery, often driven by a historical debate over its therapeutic survival benefit or concerns regarding additional surgical morbidity. Recent data have demonstrated that extended lymph node sampling does not increase postoperative complication rates (such as chylous ascites or bowel obstruction) in children, supporting its safety profile. In our patient, formal LNS was omitted due to the lack of clinical or radiological adenopathy. However, given that the final histopathological examination revealed lymphovascular invasion, the routine adoption of an LNS protocol would have provided definitive pathological nodal staging, safeguarding against potential under-staging and optimizing long-term surveillance strategies. Consistent with wider pediatric literature, local lymph node involvement in children with RCC does not carry the same heavily adverse prognostic detriment seen in adult cohorts, and long-term survival can still be achieved.^12^, ^13^, ^14^

Overall survival in pediatric RCC is highly dependent on the stage at presentation. Localized disease carries a favorable prognosis, with reported 5-year survival rates exceeding 90% for stage I–II disease, while advanced stages demonstrate significantly lower survival. Children with lymph node involvement may still achieve good outcomes, a pattern distinct from adult RCC cohorts where nodal metastasis portends poorer survival. Long-term surveillance remains essential due to the risk of late recurrence or metachronous malignancies. Emerging molecular and targeted therapies, particularly for genetically defined subtypes such as translocation RCC, represent an evolving area of research. Insights from adult RCC targeted therapy paradigms (VEGF pathway inhibitors) may inform future pediatric treatment strategies, although prospective pediatric data remain limited.^5^^,^^8^

## 4 Conclusions

Renal cell carcinoma, although rare in the pediatric population, should be considered in the differential diagnosis of children presenting with persistent or unexplained gross hematuria, particularly in older age groups. This case highlights the diagnostic challenges associated with pediatric renal masses and underscores the importance of comprehensive imaging evaluation followed by definitive histopathological confirmation. Early recognition and timely surgical intervention remain crucial, as complete surgical resection offers the best chance for favorable outcomes in localized disease. Given the distinct biological behavior and limited responsiveness of pediatric RCC to adjuvant therapies, awareness of this entity is essential to guide appropriate clinical decision-making. Continued reporting of pediatric RCC cases is necessary to enhance understanding of its presentation, optimize management strategies, and improve long-term outcomes in this rare but significant malignancy.

## CRediT authorship contribution statement

**Agung Damai Harikatang:** Visualization, Validation, Methodology, Funding acquisition, Formal analysis, Data curation, Conceptualization. **Safendra Siregar:** Writing – review & editing, Writing – original draft, Validation, Supervision, Project administration, Methodology, Investigation. **Ghani Ikhsan Majid:** Writing – original draft, Visualization, Validation, Methodology, Formal analysis, Data curation.

## Ethics declaration

Written informed consent to take part in the study and to publish the article has been obtained from all participants or their legal representatives. The privacy rights of participants have been observed.

This study included organ or tissue donors. This study includes human biological material and consent was obtained by donors, or their next of kin or legal representatives, for use in this study and for publication of the article. The samples used in this research were not sourced from executed prisoners or prisoners of conscience.

This study was performed in compliance with relevant laws, regulatory frameworks and guidelines where the research took place. Ethics committee approval was not required under relevant laws and institutional guidelines. no required in my institution

This research follows the CARE guidelines and the CARE checklist.

## Ethical statement

This case report was reviewed and approved by the Ethics Committee of Hasan Sadikin General Hospital. The study was conducted in accordance with the principles of the Declaration of Helsinki. Written informed consent for publication of the clinical details and accompanying images was obtained from the patient's legal guardian prior to manuscript submission. Patient confidentiality and anonymity were strictly maintained throughout the preparation of this report.

## Funding

I further declare that I have no financial, personal, professional, or other relationships that could be perceived as influencing the content, interpretation, or presentation of the work. No financial or non-financial interests have inappropriately influenced the conduct or reporting of this case report.

## Declaration of conflict of interest

I, Agung Damai Harikatang, as the author of the manuscript submitted to Urology Case Reports, hereby declare that there is no conflict of interest related to the preparation, submission, and publication of this manuscript.

I further declare that I have no financial, personal, professional, or other relationships that could be perceived as influencing the content, interpretation, or presentation of the work. No financial or non-financial interests have inappropriately influenced the conduct or reporting of this case report.

This declaration is provided in accordance with the journal's requirements for transparency and disclosure.
